# Clinical Impact and Outcomes of Neoadjuvant Systemic Therapy for Invasive Lobular Carcinoma

**DOI:** 10.1245/s10434-026-20130-4

**Published:** 2026-07-03

**Authors:** Genevieve A. Fasano, Jason Mouabbi, Helen M. Johnson, Heather Y. Lin, Kerollos Nashat Wanis, Susie X. Sun, Anthony Lucci, Vicente Valero, Melissa Mitchell, Yu Shen, Kelly K. Hunt, Henry Kuerer

**Affiliations:** 1 Department of Breast Surgical Oncology, The University of Texas MD Anderson Cancer Center, Houston, TX USA; 2 Department of Breast Medical Oncology, The University of Texas MD Anderson Cancer Center, Houston, TX USA; 3 Department of Biostatistics, The University of Texas MD Anderson Cancer Center, Houston, TX USA; 4 Department of Breast Radiation Oncology, The University of Texas MD Anderson Cancer Center, Houston, TX USA

**Keywords:** Invasive lobular carcinoma, Neoadjuvant systemic therapy, Neoadjuvant chemotherapy, Neoadjuvant endocrine therapy, Pathologic complete response

## Abstract

**Background:**

Studies regarding therapeutic response of invasive lobular carcinoma (ILC) to neoadjuvant systemic therapy (NST) suggest that ILC is less favorable and the prognostic impact of a pathologic complete response (pCR) is unclear.

**Patients and Methods:**

This study included 853 (NAC; *n* = 715 and NET; *n* = 138) non-metastatic classic ILC cases that received NST followed by surgery. Propensity-score matched analysis (PSMA) was performed to compare clinicopathologic groups. Survival outcomes were compared for patients with and without a pCR.

**Results:**

Most had large primary tumors with nodal metastases (89.5% cT2–T4, 64% cN+) and luminal subtype tumors (87.6%). Patients receiving NAC were more likely to be clinically node-positive (69.1% versus 34.3%; *p* = 0.0005), have grade 3 disease (18.7% versus 8.7%; *p* = 0.0082), and undergo mastectomy (84.2% versus 69.6%; *p* < 0.001) and ALND (84.9% versus 57.3%; *p* < 0.0001) compared with NET. Overall, breast and axillary nodal pCR rates were 4.9% and 9.3%, respectively. Among 155 patients who had SLNB, there was 1 axillary nodal recurrence (0.6%). The 10-year distant recurrence-free survival (DRFS) for patients with a breast pCR was 61% versus 46% for those without (*p* = 0.267). Patients with a nodal pCR demonstrated higher 10-year DRFS (49% versus 33%; *p* = 0.026) and OS compared with those without (65% versus 48%; *p* = 0.068). No survival outcome difference was seen for patients treated with NAC versus NET on PSMA.

**Conclusions:**

Nodal pCR was associated with survival outcomes in ILC and systemic therapy decision-making should be reconsidered. SLNB after NST appears to be safe and effective in ILC.

**Supplementary Information:**

The online version contains supplementary material available at https://doi.org/10.1245/s10434-026-20130-4.

Invasive lobular carcinoma (ILC) is the second most common histologic subtype of breast cancer after invasive ductal carcinoma (IDC) and displays unique features. ILC is more difficult to detect clinically and radiologically due to a linear growth pattern, and often presents with atypical symptoms such as nipple inversion, skin dimpling, or thickening.^1–4^ In addition, ILC has been shown to confer a higher risk of bilateral breast involvement, late recurrence, and development of metachronous contralateral breast cancer.^5–8^ Unlike IDC, ILC displays a distinctive metastatic pattern, more often spreading to the gastrointestinal tract, urinary tract, and ovaries.^2,9^ Population-based cancer registry data show that the incidence of ILC has increased in recent years from 6 cases per 100000 women in 1975 to 14 in 2021.^10^

Patients with ILC are more likely to have hormone receptor (HR)-positive and HER2-negative disease compared with IDC, which impacts outcomes after treatment with neoadjuvant systemic therapy (NST) since pCR is less likely among HR-positive disease.^10^ Large observational studies evaluating the rate of pathologic complete response (pCR) in response to neoadjuvant chemotherapy (NAC) in ILC and IDC have shown that ILC has lower pCR rates compared with IDC, with average pCR rates of 3–11%, compared with 10–20% for IDC.^11^ In addition, the prognostic impact of a pCR in patients with ILC has been called into question, with at least one study showing a lack of correlation with long-term outcomes.^12^ Further, early stage ILC has been shown to be responsive to neoadjuvant endocrine therapy (NET).^13^ Given these findings, there is a need to better understand the biology and therapeutic implications to elucidate the role and optimal choice and sequence of systemic agents, as NST can impact surgical management for patients with ILC.

In this study, we characterize the clinicopathologic features, treatment, and oncologic outcomes among patients with non-metastatic ILC treated with either NAC or NET and examine the breast and axillary nodal pCR rates among patients treated with NAC or NET.

## Patients and Methods

### Study Population

After institutional review board (IRB) approval, the prospectively maintained MD Anderson Cancer Center Breast Cancer Management System database was used to identify patients with non-metastatic classic ILC who received NST (NAC and NET), underwent surgery, and had at least 1 year of follow-up from 1991 to 2023. Patients with distant metastases at the time of initial diagnosis were excluded.

### Variables

Clinicopathologic characteristics and treatment information were obtained from the MD Anderson Cancer Center Breast Cancer Management System database and verified and updated from review of the electronic health record. Variables of interest included age at diagnosis, self-reported race/ethnicity, tumor grade, subtype, clinical T and N category, type of NST received (NAC or NET), breast and axillary surgery, contralateral prophylactic mastectomy, pathologic T and N category, receipt of post-neoadjuvant chemotherapy, radiation, and endocrine therapy.

### Statistical Analysis

Among patients diagnosed with non-metastatic ILC who received NST, clinicopathologic and treatment characteristics were described. Chi-squared test or Fisher exact test were used to test associations between categorical variables and Wilcoxon rank-sum test or Kruskal–Wallis test were used to detect differences for continuous variables between groups.^14^

Logistic regression models were used to assess the multivariate relationships between breast pCR or nodal pCR and patient demographic, pathologic, and clinical characteristics.^15^ A logistic regression model was obtained by first including an initial set of candidate predictor variables with a *p*-value of < 0.05 in the univariate analysis. A backward elimination was then performed using 0.05 for the significance level of the Wald chi-squared for an effect to stay in the multivariable model. Once the list of variables to be used in our final model was selected, multicollinearity between the variables was examined.

Time-to-event outcomes included distant recurrence-free survival (DRFS), time to local regional recurrence (TTLRR), disease-free survival (DRS), and overall survival (OS). DRFS was defined at the time from definitive surgery to distant recurrence or death, whichever occurred first. TTLRR was defined as the time from definitive surgery to local regional recurrence. DFS was defined as time from definitive surgery to local regional recurrence, distant recurrence, contralateral breast cancer, or death, whichever occurred first. Patients without any of these events by the time of data analysis were censored at the time of last follow-up. OS was defined as the time from initial diagnosis to death. Patients alive at analysis were censored at their time of last known contact.

The distributions of the time to event outcomes were estimated by the Kaplan–Meier method.^16^ Log-rank test was performed to test the differences in survival distributions between groups.^17^ Regression analyses of survival data based on the Cox proportional hazards (PH) model were conducted on the time to event outcomes.^18^ Martingale residuals were used to check function form of continuous variables in the Cox PH models. A multivariate Cox PH model was obtained by first including NST regardless of its *p*-value in the univariate analysis along with a set of candidate predictor variables with a *p*-value < 0.05 in the univariate analysis. A backward elimination was then performed using 0.05 for the significance level of the Wald chi-squared for an effect to stay in the model.

Propensity score matching was utilized to compare outcomes between the NAC and NET groups to reduce the impact of treatment selection bias on the estimation of treatment effect. For the model, we identified clinically relevant variables as well as factors associated with receipt of each type of NST to estimate the propensity scores. These variables included age at diagnosis and clinical N and T categories. For each matching, we identified 1:1 matching doublets for each of the two treatments, using a 5-to-1-digit greedy match algorithm.^19^ We used absolute standardized differences to assess balance in the covariates between the two treatment groups in which an absolute standardized difference less than 0.1 implies an adequate match. SAS version 9.4 and S-Plus version 8.2 were used to carry out the computations for all analyses.

## Results

### Patient, Tumor, and Treatment Characteristics

**Fig. 1:**
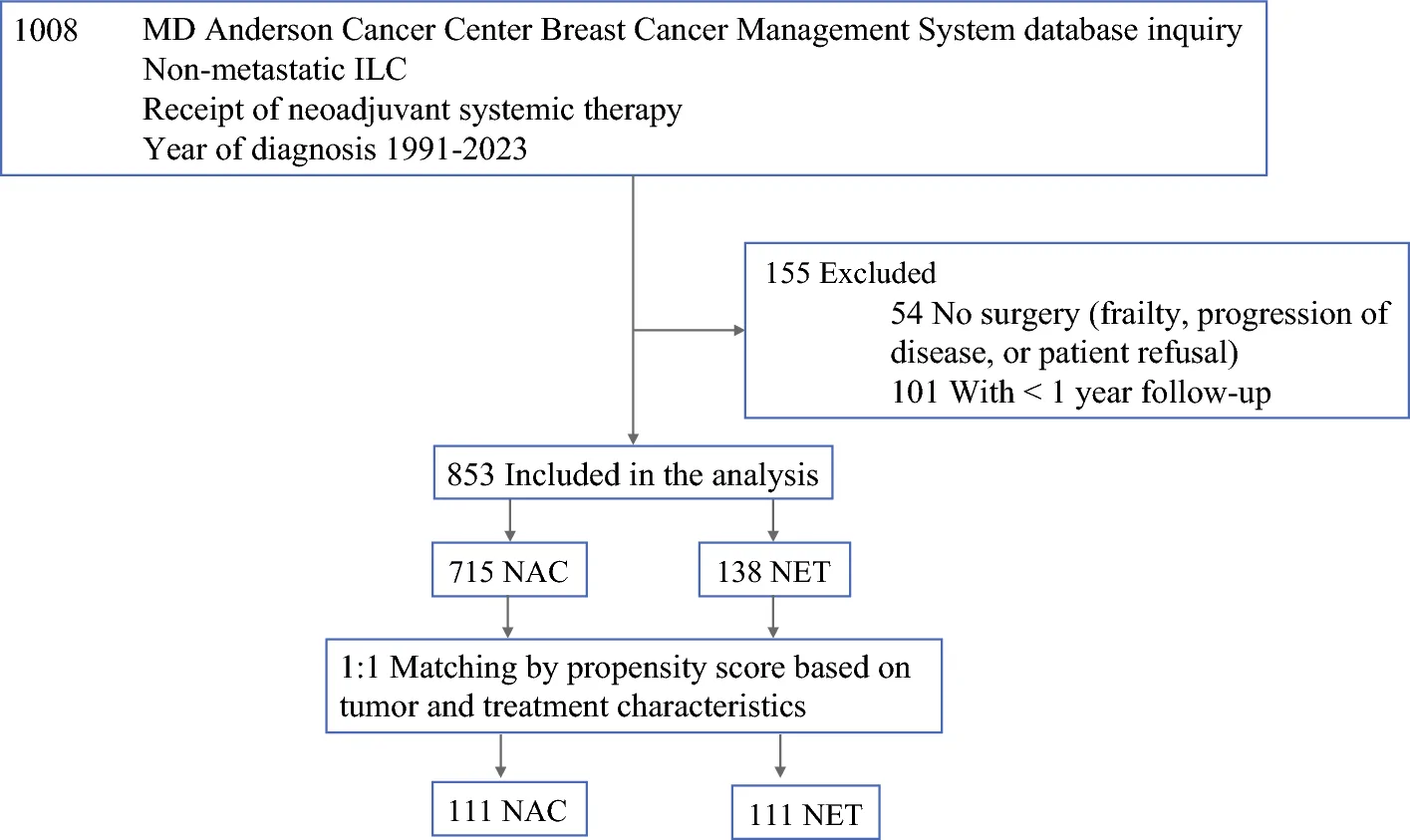
CONSORT diagram

Between 1991 and 2023, we identified 1008 patients diagnosed with non-metastatic ILC who received NST. A total of 853 patients were included in the analysis after appropriate exclusion criteria were applied (Fig. 1). Among these, 715 (83.8%) were treated with NAC, and 138 (16.2%) received NET (median length of treatment 4.5 months [IQR 3.2, 6.5 months]). Among those treated with NET, the majority (79.7%) were treated with an aromatase inhibitor; the remainder were treated with selective estrogen receptor modulators and selective estrogen receptor degraders, with or without ovarian suppression. Table 1 summarizes patient characteristics in both the NAC and NET groups in the original (unmatched) cohort. Most patients had large primary tumors and nodal metastases (89.5% cT2–cT4 tumors; 64% with biopsy-proven nodal metastasis). Patients receiving NAC were more likely to be biopsy-proven node-positive (69.1% versus 34.3%; *p* = 0.0005) and have grade 3 disease (18.7% versus 8.7%; *p* = 0.0082). Overall, 87.6% of patients had HR-positive tumors, with 9.3% having HER2+ disease and 4.1% with triple negative tumors.

**Table 1:** Patient characteristics by neoadjuvant systemic therapy received in the original cohort

Variable	Level	NAC	NET	Overall	*p*-value^1^
No. (%)		715	138	853	
Race/ethnicity	Asian/Pacific Islander	30 (4.2)	4 (2.9)	34 (4.0)	0.5707
	Black	62 (8.7)	9 (6.5)	71 (8.3)	
	Native American	1 (0.1)	0 (0.0)	1 (0.1)	
	Other	22 (3.1)	1 (0.7)	23 (2.7)	
	Hispanic	73 (10.2)	14 (10.1)	87 (10.2)	
	White	527 (73.7)	110 (79.7)	637 (74.7)	
Median age at diagnosis (years)		52	65	55	< 0.001
Grade	I	162 (24.6)	45 (32.6)	207 (26.0)	0.0082
	II	374 (56.8)	81 (58.7)	455 (57.1)	
	III	123 (18.7)	12 (8.7)	135 (16.9)	
	Not recorded	56	0	56	
T category at diagnosis	T0	1 (0.1)	0 (0.0)	1 (0.1)	0.0005
	T1	61 (9.0)	23 (17.2)	84 (10.4)	
	T2	246 (36.4)	58 (43.3)	304 (37.5)	
	T3	271 (40.1)	47 (35.1)	318 (39.3)	
	T4	59 (8.7)	6 (4.5)	65 (8.0)	
	T4d	38 (5.6)	0 (0.0)	38 (4.7)	
	Not recorded	39	4	43	
N category at diagnosis	N0	211 (30.8)	88 (65.7)	299 (36.6)	0.0005
	N1	342 (50.0)	41 (30.6)	383 (46.8)	
	N2	29 (4.2)	0 (0.0)	29 (3.5)	
	N3	102 (14.9)	5 (3.7)	107 (13.1)	
	Not recorded	31	4	35	
Subtype	HER2 unknown	52 (7.3)	2 (1.4)	54 (6.3)	0.0005
	HR+/HER2+	60 (8.4)	2 (1.4)	62 (7.3)	
	HR+/HER2−	551 (77.1)	134 (97.1)	685 (80.3)	
	HR−/HER2+	17 (2.4)	0 (0.0)	17 (2.0)	
	Triple negative	35 (4.9)	0 (0.0)	35 (4.1)	
Breast surgery	Mastectomy	602 (84.2)	96 (69.6)	698 (81.8)	< 0.0001
	Segmental mastectomy	113 (15.8)	42 (30.4)	155 (18.2)	
Axillary surgery	Axillary lymph node dissection	604 (85.9)	75 (57.3)	679 (81.4)	< 0.0001
	Sentinel lymph node biopsy	99 (14.1)	56 (42.7)	155 (18.6)	
	Not recorded	12	7	19	
Contralateral prophylactic mastectomy	No	493 (69.6)	112 (81.2)	605 (71.5)	0.0061
	Yes	215 (30.4)	26 (18.8)	241 (28.5)	
	Not recorded	7	0	7	
Pathologic T category	ypT0	41 (6.0)	2 (1.5)	43 (5.3)	0.0005
	ypT1	169 (24.8)	20 (14.7)	189 (23.1)	
	ypT2	205 (30.1)	51 (37.5)	256 (31.3)	
	ypT3	229 (33.6)	63 (46.3)	292 (35.7)	
	ypT4	26 (3.8)	0 (0.0)	26 (3.2)	
	ypTis	12 (1.8)	0 (0.0)	12 (1.5)	
	Not recorded	33	2	35	
Pathologic N category	ypN0	213 (30.6)	58 (42.0)	271 (32.5)	0.0103
	ypN1	234 (33.6)	32 (23.2)	266 (31.9)	
	ypN2	121 (17.4)	17 (12.3)	138 (16.5)	
	ypN3	129 (18.5)	31 (22.5)	160 (19.2)	
	Not recorded	18	0	18	
Adjuvant chemotherapy	No	456 (67.1)	78 (58.6)	534 (65.7)	0.0617
	Yes	224 (32.9)	55 (41.4)	279 (34.3)	
	Not recorded	35	5	40	
Adjuvant endocrine therapy	No	70 (10.2)	4 (3.0)	74 (9.0)	0.0095
	Yes	616 (89.8)	131 (97.0)	747 (91.0)	
	Not recorded	29	3	32	
Adjuvant radiation	No	99 (14.2)	38 (28.8)	137 (16.5)	< 0.0001
	Yes	598 (85.8)	94 (71.2)	692 (83.5)	
	Not recorded	18	6	24	
Pathologic complete response	Breast	40	2	42	
	Nodal	77	2	79	

^1^Chi-squared or Fisher’s exact test; *NAC* neoadjuvant chemotherapy, *NET* neoadjuvant endocrine therapy, *HR* hormone receptor

### Surgical Management

The overall mastectomy rate was 81.8%, and patients receiving NAC were more likely to undergo mastectomy than patients receiving NET (84.2% versus 69.6%; *p* < 0.0001). Sentinel lymph node biopsy (SLNB) was performed for 18.6% of the entire cohort. Axillary lymph node dissection (ALND) was more common among patients who received NAC compared with NET (85.9% versus 57.3%; *p* < 0.0001). The use of contralateral prophylactic mastectomy was 28.5% and was more commonly performed among patients receiving NAC (30.4% versus 18.8%; *p* = 0.0061) (Table 1).

### Breast and Axillary Nodal Pathologic Complete Response

The overall rates of breast and axillary nodal pCR were 4.9% (*n* = 42) and 9.3% (*n* = 79), respectively. Among patients treated with NAC, 16.1% (*n* = 76) converted from biopsy-proven node positive to node negative compared with 4.3% (*n* = 2) with NET. In the breast, the pCR rate was 5.9% (*n* = 40) for NAC and 1.5% (*n* = 2) for NET. Among patients who had a breast pCR, nearly all received NAC (95.2%, *n* = 40). Similarly, 97.5% (*n* = 77) of patients who had an axillary nodal pCR received NAC. The two patients with biopsy-proven axillary nodes that achieved a pCR after NET were treated with letrozole and both underwent sentinel lymph node biopsy. The occurrence of a breast and axillary nodal pCR was rare (2.6%, *n* = 22). On multivariate analysis, compared with patients with HR+/HER2− patients, those with HER2+ and triple negative tumors were more likely to have a breast pCR (*p* < 0.0001). The odds of achieving a breast pCR were not statistically different between patients who received NAC and those who received NET (*p* = 0.1232). Patients who were treated with NAC were more likely to achieve an axillary nodal pCR than those treated with NET (OR 7.46 [95% CI 1.36–40.9); *p* = 0.0205). Compared with patients with HR+/HER2, patients with HER2+ or triple negative tumors were more likely to achieve an axillary nodal pCR (*p* < 0.0001).

### Oncologic Outcomes

The median follow-up time was 8.5 years (95% CI 8.1–9.3). Overall, the 10-year distant recurrence-free survival (DRFS) rate was 45% (95% CI 0.41–0.5). Patients who had either a breast pCR or axillary nodal pCR demonstrated superior 10-year DFS compared with patients without pCR (breast: 61% [95% CI 0.4–0.77] versus 46% [95% CI 0.42–0.5], *p* = 0.267; nodal: 49% [95% CI 0.34–0.62] versus 33% [95% CI 0.28–0.38], *p* = 0.026). With adjustment for initial clinical nodal category, tumor grade, pathologic N category, breast surgery, and adjuvant endocrine therapy, the DRFS rates between patients receiving NAC and NET were not statistically significant (HR 1.166 [95% CI 0.855–1.591); *p* = 0.332). With adjustment for NST, tumor grade, breast surgery, and adjuvant endocrine therapy, patients who had an axillary nodal pCR had higher DRFS than patients who did not have a nodal pCR (HR 0.660 [95% CI 0.45–0.967]; *p* = 0.0331).

Overall, 13% of the entire cohort had a locoregional recurrence. At a median follow-up of 8.5 years, the cumulative incidence of local recurrence after mastectomy was 4.8%, and in-breast tumor recurrence after breast-conserving surgery was 5.8%. A nodal recurrence occurred in 2.7%, with 12 in the axillary nodes, 8 in the supraclavicular nodes, and 2 in the internal mammary nodes. After adjusting for tumor grade and adjuvant endocrine therapy, there was no difference in time to locoregional recurrence between patients treated with NAC versus NET (HR 0.804 [95% CI 0.315–2.055]; *p* = 0.6491). Among 155 patients who had SLNB alone, there was 1 axillary nodal recurrence (0.6%).

**Fig. 2:**
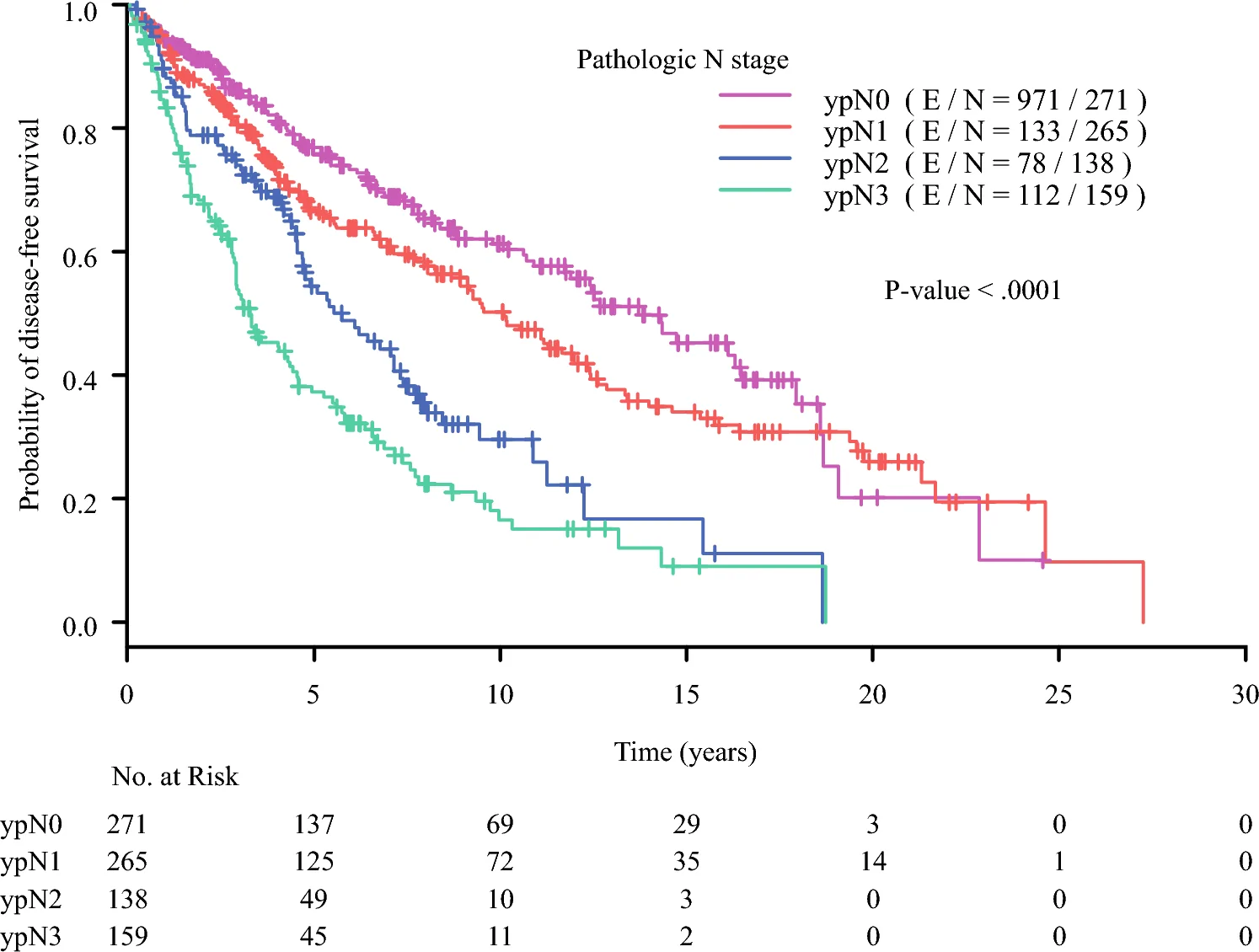
Disease-free survival in patients stratified by pathologic nodal category

The overall 10-year disease-free survival (DFS) rate was 43% (95% CI 0.39–0.47). The 10-year DFS rate was significantly higher for patients who had an axillary nodal pCR, and lower for patients with ypN3 disease (ypN0 0.61 [95% CI 0.54–0.68]; ypN1 0.50 [95% CI 0.43–0.57]; ypN2 0.30 [95% CI 0.2–0.4]; ypN3 0.16 [95% CI 0.1–0.25]; *p* < 0.0001, Fig. 2). The 10-year DFS rate for patients with a breast pCR was 56% (95% CI 0.34–0.73) compared with 44% (95% CI 0.39–0.48) for patients who did not have a breast pCR (*p* = 0.267). The DFS difference between patients receiving NAC and NET was not statistically significant (HR 1.155 [95% CI 0.851–1.566]; *p* = 0.3459).

**Fig. 3:**
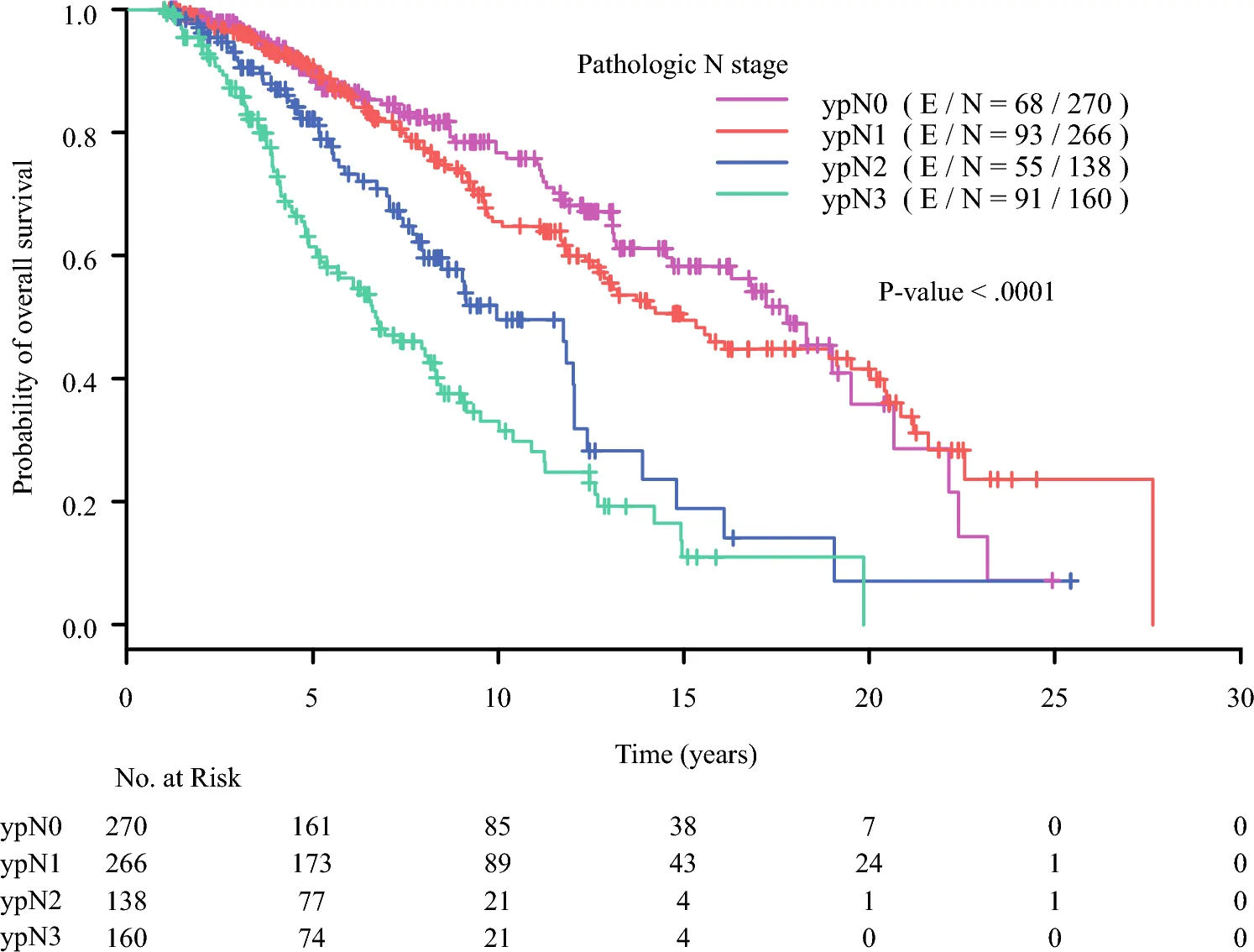
Overall survival in patients stratified by pathologic nodal category

The 10-year OS rate was 59% (95% CI 0.55–0.63) and was higher for patients with a breast pCR compared with patients without (76% [95% CI 0.53–0.89] versus 59% [95% CI 0.54–0.63]; *p* = 0.253). The 10-year OS rate was significantly higher for patients with an axillary nodal pCR, and lower for patients with ypN3 disease (ypN0 0.77 [95% CI 0.69–0.83]; ypN1 0.66 [95% CI 0.58–0.72]; ypN2 0.5 [95% CI 0.37–0.61]; ypN3 0.33 [95% CI 0.25–0.43]; *p* < 0.0001, Fig. 3). After adjustment for age at diagnosis, initial clinical nodal category, tumor grade, pathologic N category, breast surgery, and adjuvant endocrine therapy, there was no associated impact on OS for patients treated with NAC versus NET (HR 1.217 [95% CI 0.826–1.793];* p* = 0.3198).

At a median follow-up of 8.5 years, among patients not undergoing initial contralateral prophylactic mastectomy at their initial surgery, there were 8/605 (1.3%) patients who developed contralateral breast cancer.

### Oncologic Outcomes in the Matched Data

Following the 1-to-1 matching by propensity score, 111 patients in the NAC group were matched to 111 patients in the NET group. The baseline covariates between the two treatment groups in the matched data were adequately matched on the basis of the small absolute standardized differences less than 10% (Table S1). After propensity-score matched analyses, there were no significant differences in distant disease-free survival (HR 1.278 [95% CI 0.69–2.368], *p* = 0.436), time to locoregional recurrence (HR 0.8 [95% CI 0.215–2.979], *p* = 0.739), DFS (HR 1.389 [95% CI 0.758–2.546], *p* = 0.288), or OS (HR 2.286 [95% CI 0.94–5.556], *p* = 0.0681) for patients treated with NAC compared with NET (Table 2).

**Table 2:** Mortality hazard ratios of patients treated with NET versus NAC from propensity-score matched analysis

Oncologic outcome	Hazard ratio	95% confidence interval	*p*-value
DDFS	1.278	0.690–2.368	0.436
Time to LRR	0.800	0.215–2.979	0.739
DFS	1.389	0.758–2.546	0.288
OS	2.286	0.940–5.556	0.068

*NET* neoadjuvant endocrine therapy, *NAC* neoadjuvant chemotherapy, *DDFS* distant disease-free survival, *LRR* local-regional recurrence, *DFS* disease-free survival, *OS* overall survival

## Discussion

This study represents the largest single institutional experience of ILC treated with NST and includes patients treated over more than three decades. Among patients with ILC treated with NST, the occurrence of an axillary nodal pCR occurred more frequently than a breast pCR and was predictive of oncologic outcomes, as patients with an axillary pCR demonstrated increased 10-year DRFS, DFS, and OS compared with patients without an axillary pCR. Notably, no apparent benefit was detectable for patients treated with NAC compared with NET, after both multivariate analysis and propensity-score matched analysis.

Contemporary studies of NST have sought to characterize rates of pCR in women with ILC versus IDC and to determine whether pathologic response correlates with oncologic outcomes.^12,20–29^ A recent systematic review and meta-analysis of 17 studies of ILC treated with NAC included 1764 patients with ILC and 12,645 patients with IDC and showed that ILC patients had a lower pCR rate compared with IDC (5.9% versus 16.7%) as well as a lower rate of breast-conserving surgery.^29^ Loibl et al. also reported a significantly lower pCR rate for ILC compared with non-ILC (6.2% versus 17.4%) in their analysis of more than 9000 German patients with breast cancer including 1051 with ILC. Furthermore, in their analysis, pCR was not associated with distant disease-free and loco-regional disease-free survival, suggesting that the occurrence of a pCR does not correlate with oncologic outcomes.^12^ The highest overall nodal pCR rate among ILC reported in the literature to date among all subtypes is 40.9%, which was reported among tumors defined as high-risk using the 70-gene assay (Mammaprint) from the I-SPY2 trial.^30^ This is consistent with recent work by Blumencranz et al. that demonstrated that genomically high-risk tumors as defined by Mammaprint respond better to NAC and have higher likelihood of nodal downstaging.^31^ In our study, the overall rates of breast and axillary nodal pCR were 4.9 and 9.3%, respectively; in both groups, nearly all patients who achieved pCR received NAC. Similarly in our data, compared with luminal A tumors, patients with HER2+ and triple negative tumors were more likely to have a breast and nodal pCR (*p* < 0.0001). Given that patients with triple negative and HER2+ breast cancer are more likely to be treated with neoadjuvant chemotherapy, this likely contributes to the higher nodal pCR rate seen for those treated with NAC compared with NET. However, only the occurrence of an axillary nodal pCR was predictive of outcomes.

Our cohort included 138 patients with ILC treated with NET and 715 patients treated with NAC. Unlike NAC regimens where duration of treatment tends to be standardized, the duration of treatment for NET may vary. For example, in some instances, NET may be used for extended periods of up to a year, while in other scenarios, such as during the coronavirus disease 2019 (COVID-19) pandemic, NET may be offered for weeks or a few months as bridging endocrine therapy; this heterogeneity in length of treatment underscores the different goals of NET as true NST versus bridging therapy. In our study, the median length of treatment with NET was 4.5 months. Given the sample size of only 138 NET patients in our cohort overall, we did not further stratify this group into NET versus cases that were only considered bridging endocrine therapy, which is a limitation of this study. However, in future studies utilizing larger datasets, this important distinction between NET and bridging therapy should be further defined and refined in larger retrospective databases and in prospective data collection. On both multivariate analysis and propensity-score matched analysis, there were no significant differences in oncologic outcomes between patients receiving NAC compared with those receiving NET, which is similar to prior work. For example, Thornton et al. recently analyzed women with cT1–4c, cN1–3 HR+ ILC treated with NST within the National Cancer Data Base. In their analysis of nearly 6000 patients, NAC recipients had higher unadjusted 10-year OS compared with NET recipients; however, after adjustment, there was no significant difference in OS between the two groups.^32^ As genomic assays such as Oncotype DX and Mammaprint have been validated in ILC, these findings underscore the need for molecular profiling of lobular tumors to better risk stratify and select patients who may benefit most from NAC.^33–35^

Regarding surgical treatment, among 853 patients analyzed, the overall rate of breast conserving surgery was 18.2%, with most of the cohort undergoing mastectomy. This is largely a reflection of patients with larger tumors and advanced nodal disease at diagnosis. With respect to axillary management, 18.6% of the entire cohort underwent SLNB alone. Patients treated with NAC had a significantly higher rate of undergoing ALND compared with NET (85.9% versus 57.3%; *p* < 0.0001). Within the NET patients, 42.7% had SLNB as definitive axillary surgery. This is similar to current trends in axillary surgery after NST.^36^ Notably, this dataset represents the largest in the published literature to our knowledge with the longest follow-up for SLNB in patients with ILC. Among the 155 patients who did undergo SLNB as definitive axillary surgery, only one axillary nodal recurrence was observed in long-term follow-up, suggesting that among clinically and ultrasound node negative patients after NST, SLNB appears to be oncologically safe.

Overall, the rate of contralateral prophylactic mastectomy at the time of definitive surgical management was 28.5%, which is similar to national trends.^37^ Differences were seen with regard to contralateral prophylactic mastectomy among patients being treated with NAC compared with NET. Specifically, patients in the NAC group were more likely to undergo CPM compared with NET (30.4% versus 18/8%; *p* = 0.0061). The current analysis is particularly notable as only 1.3% of patients developed contralateral breast cancer, suggesting that contralateral prophylactic mastectomy among high-risk ILC receiving NST does not appear to be warranted.

### Limitations

The main limitation of this study is the retrospective nature, although there are important strengths regarding a very large dataset of a relatively less common form of breast cancer treated utilizing uniform treatment algorithms within a large comprehensive cancer center. Additionally, while this represents the largest single-institutional study, patients were treated over three decades, which is valuable due to length of follow-up; however, during this time, indications for systemic therapy have evolved.

## Conclusions

In this large, single-institutional study, clinicopathologic and treatment characteristics as well as the response of ILC to NST for patients receiving either NAC or NET were investigated. The findings from this large study demonstrated that an axillary nodal pCR is an important prognostic factor in survival outcomes for patients with ILC treated with NST. Given the lack of benefit seen for NAC compared with NET in survival outcomes, upfront molecular profiling at the time of diagnosis may be warranted to optimally risk stratify patients and optimize NST regimen selections. The low rate of locoregional recurrence for patients undergoing SLNB alone as definitive axillary surgery supports deescalation of axillary surgery, even among this high-risk subset.

## Supplementary Information

Below is the link to the electronic supplementary material.

Supplementary file1 (DOCX 13 KB)
