# Supplementary material for: Clinical Impact and Outcomes of Neoadjuvant Systemic Therapy for Invasive Lobular Carcinoma
Source: Ann Surg Oncol. 2026 Jul 3;33(11):9609–17. doi: 10.1245/s10434-026-20130-4 (PMC13631023; doi:10.1245/s10434-026-20130-4)
Supplement: Supplementary file 1 [file 10434_2026_20130_MOESM1_ESM.docx]

**Table S1.** Patient characteristics by neoadjuvant systemic therapy received in the matched data

| **Variable** | **Level** | **NAC** | **NET** | **Overall** | **Absolute standardized difference** |
| --- | --- | --- | --- | --- | --- |
| No. (%) |  | 111 | 111 | 222 |  |
| Median age at diagnosis (years) |  | 62 | 63 | 62.5 | 0.031 |
| Clinical T category | T1 | 12 (10.8) | 16 (14.4) | 28 (12.6) | 0.109 |
|  | T2 | 47 (42.3) | 48 (43.2) | 95 (42.8) |  |
|  | T3 | 47 (42.3) | 43 (38.7) | 90 (40.5) |  |
|  | T4 | 5 (4.5) | 4 (3.6) | 9 (4.1) |  |
| Clinical N category | N0 | 73 (65.8) | 68 (61.3) | 141 (63.5) | 0.108 |
|  | N1 | 34 (30.6) | 39 (35.1) | 73 (32.9) |  |
|  | N3 | 4 (3.6) | 4 (3.6) | 8 (3.6) |  |
